# Evaluating Religious Influences on the Utilization of Maternal Health Services among Muslim and Christian Women in North-Central Nigeria

**DOI:** 10.1155/2016/3645415

**Published:** 2016-02-24

**Authors:** Maryam Al-Mujtaba, Llewellyn J. Cornelius, Hadiza Galadanci, Salome Erekaha, Joshua N. Okundaye, Olusegun A. Adeyemi, Nadia A. Sam-Agudu

**Affiliations:** ^1^Institute of Human Virology Nigeria, Plot 252 Herbert Macaulay Way, Abuja 900246, Nigeria; ^2^School of Social Work, University of Georgia, 279 Williams Street, Athens, GA 30602, USA; ^3^Department of Obstetrics and Gynaecology, Aminu Kano Teaching Hospital, PMB 3452, Kano 700231, Nigeria; ^4^Department of Health Promotion and Education, University of Ibadan, Ibadan 200284, Nigeria; ^5^School of Social Work, University of Maryland Baltimore County, 1000 Hilltop Circle, Baltimore, MD 21250, USA; ^6^Division of Epidemiology and Prevention, Institute of Human Virology, University of Maryland Baltimore, 725 West Lombard Street, Baltimore, MD 21201, USA

## Abstract

*Introduction*. Uptake of antenatal services is low in Nigeria; however, indicators in the Christian-dominated South have been better than in the Muslim-dominated North. This study evaluated religious influences on utilization of general and HIV-related maternal health services among women in rural and periurban North-Central Nigeria.* Materials and Methods*. Targeted participants were HIV-positive, pregnant, or of reproductive age in the Federal Capital Territory and Nasarawa. Themes explored were utilization of facility-based services, provider gender preferences, and Mentor Mother acceptability. Thematic and content approaches were applied to manual data analysis.* Results*. Sixty-eight (68) women were recruited, 72% Christian and 28% Muslim. There were no significant religious influences identified among barriers to maternal service uptake. All participants stated preference for facility-based services. Uptake limitations were mainly distance from clinic and socioeconomic dependence on male partners rather than religious restrictions. Neither Muslim nor Christian women had provider gender preferences; competence and positive attitude were more important. All women found Mentor Mothers highly acceptable.* Conclusion*. Barriers to uptake of maternal health services appear to be minimally influenced by religion. ANC/PMTCT uptake interventions should target male partner buy-in and support, healthcare provider training to improve attitudes, and Mentor Mother program strengthening and impact assessment.

## 1. Introduction

Of the 21 sub-Saharan African Global Plan priority countries, Nigeria has had the lowest PMTCT coverage rates and highest PMTCT service delivery gap. With approximately 190 000 HIV+ women giving birth yearly in Nigeria [1], PMTCT service coverage is only 27.0%–30.1% for eligible women [1–3]. The country is also responsible for 25% of the global PMTCT gap [1]. And after more than 10 years of PMTCT country-wide program implementation, the Mother-to-Child Transmission of HIV (MTCT) rate among HIV-positive Nigerian women is 26% [1, 4]. Since the Nigerian PMTCT program is integrated with antenatal care (ANC) services, the uptake of ANC is directly related to the uptake and success of PMTCT program. ANC and PMTCT programs are integral components of maternal health services. Maternal health encompasses the health of a woman during pregnancy, childbirth, and postpartum [5].

Poor or no ANC and PMTCT care impacts on maternal and infant outcomes such as maternal mortality, infant mortality, and MTCT. In Nigeria, there are over 7 million live births annually [2]. Only 61% of pregnant Nigerian women attend the WHO-recommended 4 ANC visits, and less than 60% of births are attended by a skilled birth attendant [6]. In rural areas, where two-thirds of Nigerians reside [7], ANC visit attendance is poor (51.6–56.8%), compared to urban areas (75.9–82.0%) [6, 7]. Likewise, births attended by a skilled attendant are fewer in rural areas (35.4–46.6%) compared to urban areas (71.4–86.0%) [6–8]. Almost half (48.8%) of ANC nonusers stated they could not access clinic services because clinics were too far away, and this is expressed more by rural women (52.0%) than their urban counterparts (33.7%) [9]. Infant mortality rate in Nigeria is 58 per 1,000 live births [6]. However, rural areas experience 63 deaths per 1,000 live births, with 46 deaths per 1,000 live births in urban areas [6].

Low rates of maternal health service utilization in sub-Saharan Africa (SSA) have been linked to women's socioeconomic dependency on men, and unequal gender relations arising from religious and cultural influences [10–12]. Spirituality and faith-based practices play an integral role in coping with psychological difficulties in illness and in health-seeking behaviors [13]. Religious influences may therefore explain some of the disparities in uptake of available healthcare services within or between some populations.

As of 2015, Nigeria has a population of over 182 million people [14]. The country has evolved into a Christian-dominated South (with 84.4% Christians) and a Muslim-dominated North (with 81.8% Muslims), while the North-Central middle belt has a more equitable distribution of the major religious faiths (42.0% Muslims, 56.0% Christians, and 2% other religions) [7, 15]. Healthcare-seeking behavior with respect to ANC differs greatly between the Muslim-dominated North and the Christian-dominated South. The proportion of pregnant women attending the WHO-recommended 4 antenatal visits is highest in the South (76.8–89.0%), followed by North-Central (66.0–76.0%), and least in the North (35.5–51.9%) [6, 8, 9]. Likewise, childbirths attended by a skilled birth attendant are highest in the South (73.4–78.8%), followed by North-Central (46.5–67.2%), and again lowest in the North (16.1–27.8%) [6, 8]. Within the country, births with no attendant (skilled or unskilled) present have been highest in the North (77.2–86.5%), less in North-Central (7.2–13.4%), and least in the South (6.3–9.5%) [8, 16].

Maternal health outcomes also portray staggering North-South differences. The national Maternal Mortality Ratio (MMR) is 560 per 100,000 live births compared to the global average of 210 per 100,000 [17]. The North, with MMR of 1,000 per 100,000 live births, disproportionately contributes to this alarming statistic compared to the South with MMR of 300 per 100,000 live births [18]. Likewise, Infant Mortality Rates are worse in the North (77.3 per 1,000), compared to the South (67.0 per 1,000) [8]. The dichotomy between the significantly Muslim North and Christian-dominated South with regard to maternal service uptake and maternal-infant outcomes suggests underlying religious influences.

There is a paucity of literature from Nigeria and other SSA countries on associations between religious beliefs and uptake of maternal health services among Christian women. An Ethiopian study reported that orthodox Christians do rely on their spirituality and faith-based practices in health-seeking behavior and in coping with illness [13].

The extent to which religious law or teachings influence health-seeking behavior might differ between and within countries. For example, in the Middle East and North Africa, a Muslim woman is required to have her husband or a male relative to accompany her outside the home [19, 20]. In addition to that, in Afghanistan, a Muslim woman needs her husband's permission to leave the house even in the case of an emergency [19]. In Northern Ghana, even though Muslim women prefer skilled birth attendants, healthcare workers' obliviousness, or insensitivities to their religious obligations, practices, and maternity needs, significantly hinders their ability to use these services [12]. Therefore, poor uptake of skilled delivery services in this regard is not necessarily due to religious influences but healthcare worker knowledge and attitudes.

A few studies have investigated faith-based influences on healthcare utilization among Muslim Nigerian women. Previous studies in Northern Nigeria described faith-related factors that were barriers to Muslim women's use of maternal health services: having to obtain permission from significant others [9, 21, 22], parents, guardians, cultural or religious leaders [9], and unwillingness to be attended to by a male healthcare provider [9, 22]. This dynamic may cause women to have reduced autonomy and decision-making power concerning their health, which extends to uptake of maternal health services [12, 19]. Given the more balanced representation of Christians and Muslims, the North-Central region affords a useful opportunity to examine the influences of religion on health-seeking behavior within one accessible geographical area. It is also important to assess religious influence on HIV service uptake in the North-Central region because it has some of the highest HIV-burden states in the country. Out of Nigeria's 37 states (including the Federal Capital Territory), North-Central has a 3.4% HIV prevalence in the general population, higher than the national average of 3.0% [3]. Within that region, Nasarawa and FCT present seroprevalences of 8.1% and 7.5%, respectively, and these are among the top 5 states with highest HIV prevalence in the country [3]. The dual advantage of conducting such a study in the North-Central region is that it presents a robust mix of Muslims and Christians living in proximity to each other in high HIV-burden communities [7]. Given the poorer maternal health service uptake indices in Muslim-dominated Northern Nigeria, it is important to evaluate for religious influences on these phenomena in North-Central Nigeria, where HIV burden is high and uptake has been historically low. The purpose of this study was to evaluate for and compare and contrast faith-related barriers to utilizing ANC and PMTCT services among Muslim and Christian women in North-Central Nigeria.

## 2. Materials and Methods

### 2.1. Study Setting

The venues for this qualitative study were one urban community and 6 periurban and rural Primary Healthcare Centers (PHCs) located in the Federal Capital Territory (FCT) and Nasarawa State in North-Central Nigeria. The 6 sites were selected from a list of 26 study-eligible PHCs assessed for a large implementation research study conducted in rural and hard-to-reach areas of the aforementioned states (see Section 2.2). The 2006 National Census survey reported populations for FCT and Nasarawa at 1,406,239 and 1,869,377, respectively [23]. As of 2013, the population of Nasarawa comprised 56.7% Christians and 41.1% Muslims [8], while FCT comprised 68.0% Christians and 27.4% Muslims [7]. General population HIV prevalence in Nasarawa and FCT is 8.1% and 7.5%, respectively [7]. The most recent antenatal HIV seroprevalence survey reported 7.5% for Nasarawa and 8.6% for FCT, against a national antenatal seroprevalence of 4.1% [24].

### 2.2. Study Population

Study population included pregnant ANC attendees, HIV-positive women, and young women of childbearing age. Focus Group Discussions (FGDs) were conducted as part of the MoMent Nigeria study, a Canadian government-funded and World Health Organization-supported PMTCT implementation research project under the 6-study, 3-country INSPIRE initiative [25]. MoMent Nigeria investigates the impact of trained peer HIV-positive counselors (Mentor Mothers) on PMTCT outcomes for HIV-positive women and HIV-exposed infants [26]. MoMent's formative aspect explored barriers to PMTCT access and uptake, and acceptability of Mentor Mothers as a viable intervention [26]. Participants from the PHCs were first approached as they attended clinic or were contacted by phone by PHC healthcare workers and briefly informed of the study. Young females were recruited from the National Youth Service Corps; they were providing 12 months of community service in FCT and in Nasarawa. Given the staff profiles of the study PHCs and other healthcare facilities in the surrounding study communities, all study participants had been exposed to both male and female Sexual and Reproductive Health or maternal service providers. Women 18 years of age and over were recruited on a rolling basis according to the target focus group, for example, ANC attendees or HIV-positive women; no other specifications were applied for recruitment. Recruitment was stopped once a target of 10 women had been reached for each FGD. Interested participants showed up for the FGD on the appointed date. The study was fully explained and consent sought by study staff. Written participant consent was obtained prior to initiation of all the FGDs. Participants received refreshments and reimbursements commiserate with transport costs applicable to the day of the FGD only. The qualitative study time period was December 2012 to April 2013.

### 2.3. Ethical Approval

Ethical approval was granted by the Institutional Review Boards of the Institute of Human Virology Nigeria and the University of Maryland, Baltimore.

### 2.4. Data Collection

Two FGDs were conducted among Mentor Mothers, 2 among pregnant ANC clinic attendees, 2 among mother-to-mother (M2M) HIV support group members, and 1 with young women, totaling 7 FGDs. An interviewer-administered form was used to capture participants' sociodemographic information such as religious affiliation, age, place of residence, marital status, and parity. Religious affiliation data was collected only after consent was provided and as such the focus groups were a mixture of both Muslim and Christian women. The FGDs were guided by semistructured questionnaires organized thematically as follows: barriers to uptake of ANC services (cost, distance, quality of ANC service, and attitude of healthcare providers), women's views and experiences as members of M2M groups, and/or being Mentor Mothers, use of unconventional healthcare services or remedies, stigma related to HIV+ status, gender preference for a healthcare provider, and acceptability of Mentor Mother services. FGD facilitators comprised two Social Science professors as well as health professionals (doctors, nurses) and graduate students trained to conduct FGDs. A moderator and comoderator facilitated all FGDs along with at least one observer documenting synergistic group effects and nonverbal cues. Sessions were conducted in English and/or Hausa (the dominant language of the study communities). Bilingual (English and Hausa) facilitators were involved in the conduct, transcription, and analysis of all the FGDs. To maintain anonymity and establish a conducive atmosphere for discussion, participants used self-chosen aliases for each FGD. Each FGD was audio-recorded and lasted approximately 60–90 minutes.

### 2.5. FGD Transcription and Data Analysis

Audio recordings were transcribed verbatim; Hausa sessions were transcribed into English by bilingual study staff. FGD transcripts were assigned to 4 groups of 2 coders each. Coders were the same trained individuals who facilitated and transcribed the FGDs. Each group member independently hand-coded their assigned transcript by reviewing each line, phrase, and paragraph to identify the initial key themes. Subsequently, each coding group met separately and then with all other groups, for review and merging of independently analyzed and coded transcripts into a final document. This stage was succeeded with a joint review of each of the 5 groups' finalized transcripts by the entire team (a panel of 10 researchers from the 4 groups, facilitated by two Social Science professors). In this validation process, codes and themes were examined for content within the context of the document and thematically in relation to the overall interview guide. The resultant data were combined into one matrix to develop visual charts of the words and phrases that represented the themes discovered during analysis. In order to protect the privacy of the respondents and organizations, names of persons and institutions were deleted in the final report.

## 3. Results

### 3.1. Sociodemographic Characteristics of Study Participants

A total of 68 women participated in the 7 FGDs. Study participants resided in 25 different communities from within and up to 105 km away from the study venue/PHC location. Approximately 84% of participants resided in rural communities, while 16% were urban/periurban residents. There were more Christian participants (49/68, 72.1%) compared to Muslims (19/68, 27.9%). Details of participant characteristics are presented in Table 1.

### 3.2. Focus Group Discussion Findings

Three themes emerged from the 7 FGDs: participants' views on clinic-based ANC and delivery versus other options, preference for healthcare provider gender and religion, and the acceptability of Mentor Mothers (MMs) as a PMTCT service.

#### 3.2.1. Utilizing the Healthcare Facility for ANC and Delivery Care

A few women reported prior home deliveries; however, these were not due to choice; the deliveries were imminent. A preference for facility-based deliveries, especially amongst HIV-positive women, emerged. Stated barriers to utilizing facility-based services (ANC and PMTCT) were living far away from clinics and unaffordable transportation fees: “*We live in the village, so to come to clinic is very hard for me*”. M2M, G6. “…* if you advise them, they will say no money to come to hospital or if they ask their husband they don't have money so they are missing their appointment date so we have that challenge for our side or so*.” - MM, G2.

Nonavailability of male partners to accompany/transport women to the clinic for services also emerged as a barrier. Even when a woman planned a facility delivery, she may have to resort to home delivery if her partner was not available to transport her to the clinic at the onset of labor. “*Yes. I have delivered at home. My first-born was delivered at home. Well, I delivered at home because my husband was not around. I was the only one that was at home. And I had one midwife around my house so my aunt went and called her. But my second born was delivered in the hospital*….* because I know the importance of delivering in the hospital; they will take care of the baby and take care of me. They will know what is wrong with me; either I'm going to bleed or not going to bleed; what do I need at that particular time or what does my baby need at that particular time. And I don't advise anybody to give birth at home.*” - MM, G1.

All respondents preferred hospital delivery to home delivery. For those who were HIV-positive, knowing their status during pregnancy also encouraged them to deliver at the facility: “*One reason why some of us decide to come to the clinic instead of delivering at home is when you consider the risk. One thing is so many of us don't even find out about our status until we are pregnant.*” - M2M, G6.

Participants also articulated preference for clinic-based ANC. However, male partners' opinions could supersede a woman's preference for and utilization of skilled ANC: “*I delivered mine at home, because one man lives on our road and he said he is a doctor and so anytime I'm sick and even when I get pregnant my husband will not let me go to the hospital….Yes. He will tell me to go to the man's place; because the man is their relative.*” - M2M, G5.

#### 3.2.2. Healthcare Provider Preference

The majority of participants did not have a healthcare provider gender preference; they were simply concerned with receiving available services expeditiously. “*Any doctor that is on duty will check me.*” - MM, G1.* “Either male or female; personally, I don't care.*” - MM G1. “*I can go with anyone, male or female.*” - Young woman, G7. The women were not concerned with the gender or religious affiliation of the healthcare provider available to attend to them. Their main concern was for their own health as well as that of the unborn infants: “*What I want to say, l don't care who is there. As you're struggling for your life, the baby is struggling too. Whoever is there, make the person just*…* deliver the baby from them.*” - ANC attendee, G4. The majority of these women were open to receiving healthcare services, especially deliveries, from any healthcare provider available, male or female, Muslim or Christian. “*Will I say because I didn't see any woman there, they will not check me? They will check me now.*” - Young woman G7. “*Are we not the ones that gave birth to men? Any doctor that is on duty will check me*…* Either Muslim or Christian is all the same…Either male or female.*” - M2M, G6.

However, the women did express preference for a healthcare provider with an accommodating attitude. “*We prefer Aunty* (specific PHC healthcare worker)* to consult us because she is more considerate and she cares about us.*” - M2M, G5* “She is a mother to all.*” - M2M, G5.

Young women specifically preferred male healthcare providers because they considered males to be more patient and understanding than their female counterparts: “*I normally prefer male doctors when I go the hospital because normally, they give me more elaboration. They explain things better.*” - Young woman, G7.

“*Men. They are more patient than females.*” - Young woman, G7.

The choice of a certain gender therefore was not based on religious preference but rather professional attitude.

#### 3.2.3. Acceptability of Mentor Mothers as a PMTCT Service

Responses from Mentor Mothers indicated that HIV-positive women were generally accepting and responsive to their services. Furthermore, Mentor Mothers identified two important factors necessary for a successful mentorship: mentors' disclosure of their own HIV status to their mentees and a cordial, respectful Mentor Mother. Religious affiliation did not emerge as a barrier for acceptability of Mentor Mothers. “*For my own, since I started this MM, I have never seen a challenge because of the way I live with my people. I normally joke with them. Everybody knows me. I even tell them my status even in a church.*” - MM, G2. “*It is a challenge when the person don't know your status so if you are counselling and you don't disclose, it is a problem*” - MM, G2. “*My own experience as a Mentor Mother is very friendly. In fact, we relate as sisters in the facility.*” - MM, G1. However, Mentor Mother home visits, and mentor-mentee interaction in the community setting are not as robust as in the clinic, because of the stigma associated with HIV-positive status, and frequent interaction with a known HIV-positive individual. The community-level stigma appeared to be religion-independent. “*Yes. You know most of them here, they don't* …* out of 100, I think it is only 25% that allow us to go to their house—which is zero. Yes. 75% don't want us to come. Because maybe they're having partners at home* (co-wives)* and the partners maybe have not been tested….*” - MM, G1.

On the other hand, Mentor Mothers indicated that women who had overcome the fear of HIV-related stigma were comfortable with Mentor Mother home visits. Again, religion did not appear to affect the development, or overcoming of the fear of stigma in associating with a Mentor Mother. “*She says that yes, they faced that* (fear of stigma)* but it was more previously. But now it is just like it's the order of the day. Most people have it* (HIV)* so it's not a big deal any more, especially in her own community. So once she goes to a house, they don't normally care whether they recognize her as positive or not because ultimately, they will still refer her*—*she will still go to a health facility and whatever it is, anyhow, people will get to know that she is positive. So, it's not really a big problem. Before it used to be a problem but now it is going down.*” - MM, G1.

Respondents across groups also acknowledged that MMs would be useful, especially for new mothers. “*Yes, especially if it is your first time* (pregnancy or motherhood);* it will be confusing and scary. So it is good to have somebody who has the experience.*” - Young Woman, G7.

Respondents were generally willing to accept the services of Mentor Mothers as “helpers” whether in a HIV or non-HIV context. However, they expressed preference for the services of trained Mentor Mothers to untrained female or male relatives. This was due to instances where friends and relatives gave unhelpful lay advice based on traditional myths, which could be confusing.* “Sometimes, you meet your neighbor and she will be telling you that you should not be drinking malt, akamu *(cornmeal porridge)* or tea—that it will be making the baby to be fat. Or sometimes, this cold water. That it will make the baby to be relaxed inside your womb. The time the baby is supposed to come out, it will still remain there… that's why it's good to have all these people that are well-trained.”* - ANC attendee, G3.

## 4. Discussion

Among the largely mixed groups of Muslim and Christian women in our study population, religion did not appear to influence choices made towards healthcare facility patronage. On the contrary, the preference across both Muslim and Christian participants was to use healthcare facilities for maternal services. As stated in a Hadith (the records of sayings and preaching of the Prophet Muhammad Peace be Upon Him): “*A woman should not travel except with a Dhu-Mahram* (her husband or a man with whom that woman cannot marry at all according to the Islamic Jurisprudence),* and no man may visit her except in the presence of a Dhu-Mahram*.” [27]. However, examination of the barriers to maternal service uptake among our Muslim participants did not specifically draw out this edict or compliance to it, as a barrier. There are no specific Christian correlates in the Bible with respect to women's travel and requirements for accompaniment by a male. However, interpretations of Biblical writings by Christian religious leaders as well as Christian study participants could have resulted in conclusions similar to the quoted Hadith; however, this was not identified in the study. Studies in Northern Nigeria have however shown that less than a third of husbands make necessary provisions for their wives' maternal care and only about a quarter make provision for their wives' transportation in relation to these services [28]. Husbands not making the necessary provisions (financial and situational) for their wives to utilize maternal health services during their absence may be contributing to poor uptake, and not necessarily religious law.

A prior study identified geographical distance and transportation fees to clinic as the top two barriers to ANC access in Nigeria [9], and these barriers were more reflected among Muslim women compared to Christian women [9, 21]. These findings corroborate with those of our study and emphasize the need to make maternal health services geographically accessible in rural areas. When skilled maternal healthcare services are delivered at local posts within the community, service uptake increases exponentially [21, 29]. With regard to HIV, however, community-level stigma may cause HIV-positive women to seek ANC and PMTCT services far from their own community, where they may be less well known.

With respect to provider gender preference among women, our study findings deviate from what has been previously reported in Nigeria and other countries, where female providers were preferred [9, 12, 19, 20]. In our study, healthcare provider gender preference was not an issue with regard to religion, whether the respondents were HIV-positive or not. Even when cited, the gender preference was due to better professional mannerism among male providers. Specifically, this male gender preference was cited among the young women's group (G7). Our study participants' preference for healthcare providers seems to transcend religious affiliations and is based on a respectful, caring provider attitude. These findings are in concordance with the views of Muslim women in Ghana, who preferred healthcare providers exhibiting religious tolerance while providing care [12]. Likewise, women in rural Democratic Republic of the Congo, India, Nigeria, and Uganda frequently turned to traditional birth attendants over facility-based health providers because they were considered more familiar, friendlier, and more culturally knowledgeable [10]. It is important to note that women were willing to forgo the expected social comfort and familiarity of being attended to by a female healthcare provider for a male healthcare provider with a positive professional attitude if the female workers were rude or disrespectful. This is affirmed by the fact that, in Nigeria, poor attitude and unprofessional conduct of healthcare providers were a significant reason why women did not seek maternal health services [9].

Mentor Mother services were acceptable among all groups including HIV-positive women. However, community stigma associated with a HIV-positive status, and not religious affiliation of the mentor or mentee, limited Mentor Mothers' abilities to counsel women in community or home settings.

## 5. Conclusion

In this study among women in rural North-Central Nigeria, we found little to support a significant role for Christian or Islamic religious beliefs in influencing maternal service uptake. This was an unexpected finding; however, it does not necessarily mean that more subtle religious influences are absent in this context. The one religious law that could be extrapolated and linked to poor uptake was the requirement of Muslim women to be accompanied by their husbands away from home. However, this particular Hadith's saying was not mentioned or referred to by any of the Muslim participants during our study. Overall, barriers to utilizing maternal health services (geographical distance to facility, transportation cost, healthcare provider attitude, and gender roles with regard to decision-making) appear to be independent of religious influences. Our findings suggest that behavior and attitude with regard to maternal health service utilization in our North-Central study communities are similar amongst Muslims and Christians. In other words, religious influences on ANC and PMTCT service uptake appear to be more similar than different in our North-Central study communities where there is more equitable representation of both religions compared to the core North or South.

To encourage utilization of available maternal health services, important strategies to consider include advocating to and educating male partners on the importance of supporting access to and payment for facility-based maternal services without undermining their traditional decision-making roles. Where necessary, alternate family members should be nominated to facilitate pregnant women's attendance at facility appointments or deliveries if a husband was unavailable. In addition, healthcare providers especially in rural areas should be made aware of the impact of negative or disrespectful attitudes to the success of ANC and PMTCT programs in Nigeria. Training targeting attitudinal changes and the adoption of professional behavior are sorely needed among rural-based healthcare workers, especially females, because they make up the gender majority of healthcare workers that clients are exposed to at PHCs. Finally, high-level, across-group acceptability of Mentor Mothers signals that the uptake of their peer mentoring services could improve service uptake and retention among HIV-positive women along the PMTCT cascade, regardless of their religious affiliations.

## Figures and Tables

**Table 1:** FGD participant characteristics (^a^data not available).

	G1	G2	G3	G4	G5	G6	G7	All groups
	MM1	MM2	ANC1	ANC2	M2M1	M2M2	Young women	G1–G7
	*N* = 8	*N* = 10	*N* = 10	*N* = 10	*N* = 10	*N* = 9	*N* = 11	*N* = 68
Mean age (SD)	28.6 (±5.0)	33.3 (±3.4)	—^a^	—^a^	29.5 (±6.8)	28.1 (±2.1)	25.1 (±3.2)	30.0 (±5.0)
Other characteristics: *N* (%)								
Location of FGD venue	Rural	Rural	Rural	Rural	Rural	Rural	Urban	
Religion								
Christianity	0 (0.0)	9 (90.0)	8 (80.0)	8 (80.0)	6 (60.0)	8 (88.9)	10 (90.9)	49 (72.1)
Islam	8 (100.0)	1 (10.0)	2 (20.0)	2 (20.0)	4 (40.0)	1 (11.1)	1 (9.1)	19 (27.9)
No response	0 (0.0)	0 (0.0)	0 (0.0)	0 (0.0)	0 (0.0)	0 (0.0)	0 (0.0)	0 (0.0)
Marital status								
Single	0 (0.0)	0 (0.0)	0 (0.0)	0 (0.0)	0 (0.0)	0 (0.0)	10 (90.9)	10 (14.7)
Married	8 (100.0)	10 (100.0)	10 (100.0)	10 (100.0)	7 (70.0)	9 (100.0)	1 (9.1)	55 (80.9)
Polygamous marriage	2 (25.0)	0 (0.0)	0 (0.0)	0 (0.0)	2 (28.6)	0 (0.0)	0 (0.0)	4 (7.3)
No response	0 (0.0)	0 (0.0)	0 (0.0)	0 (0.0)	3 (30.0)	0 (0.0)	0 (0.0)	3 (4.4)
Living children								
None	0 (0.0)	0 (0.0)	3 (30.0)	7 (70.0)	0 (0.0)	1 (11.1)	10 (90.9)	21 (30.9)
1-2	4 (50.0)	2 (20.0)	4 (40.0)	3 (30.0)	4 (40.0)	5 (55.6)	1 (9.1)	23 (33.8)
3-4	4 (50.0)	6 (60.0)	3 (30.0)	0 (0.0)	3 (30.0)	1 (11.1)	0 (0.0)	17 (25.0)
>4	0 (0.0)	2 (20.0)	0 (0.0)	0 (0.0)	0 (0.0)	2 (22.2)	0 (0.0)	4 (5.9)
No response	0 (0.0)	0 (0.0)	0 (0.0)	0 (0.0)	3 (30.0)	0 (0.0)	0 (0.0)	3 (4.4)
Formal education								
None	1 (12.5)	0 (0.0)	1 (10.0)	0 (0.0)	0 (0.0)	0 (0.0)	0 (0.0)	2 (2.9)
Primary	4 (50.0)	2 (20.0)	4 (40.0)	1 (10.0)	3 (30.0)	2 (22.2)	0 (0.0)	16 (23.5)
Secondary	2 (25.0)	6 (60.0)	3 (30.0)	4 (40.0)	3 (30.0)	7 (77.8)	0 (0.0)	25 (36.8)
Tertiary	1 (12.5)	2 (20.0)	2 (20.0)	5 (50.0)	1 (10.0)	0 (0.0)	11 (100.0)	22 (32.4)
No response	0 (0.0)	0 (0.0)	0 (0.0)	0 (0.0)	3 (30.0)	0 (0.0)	0 (0.0)	3 (4.4)
Language of highest fluency								
English	0 (0.0)	0 (0.0)	1 (10.0)	0 (0.0)	0 (0.0)	0 (0.0)	11 (100.0)	12 (17.6)
Hausa	8 (100.0)	1 (10.0)	3 (30.0)	1 (10.0)	7 (70.0)	1 (11.1)	0 (0.0)	21 (30.9)
Igbo	0 (0.0)	0 (0.0)	1 (10.0)	3 (30.0)	0 (0.0)	3 (33.3)	0 (0.0)	7 (10.3)
Others	0 (0.0)	4 (40.0)	5 (50.0)	6 (60.0)	3 (30.0)	5 (55.6)	0 (0.0)	23 (33.8)
No response	0 (0.0)	5 (50.0)	0 (0.0)	0 (0.0)	0 (0.0)	0 (0.0)	0 (0.0)	5 (7.4)

## Study Limitations

This study has some limitations. First, there were disproportionately more Christian than Muslim women participants represented, partly due to the fact that the study did not specifically target equal proportions of Christians versus Muslims. This in itself could reflect that access to and/or utilization of healthcare facility services may be lower among Muslim women, even if not necessarily due to religion itself. Our conclusion that health behaviors are similar among Christians and Muslims in North-Central Nigeria may have been modified if more Muslim women were represented in our study sample. Second, some study participants were recruited directly from clinics, for instance, ANC attendees, implying that they already had preference for facility-based services over alternate or home-based care. Furthermore, the study also did not evaluate for the socioeconomic status of Muslim versus Christian women. This may have guided the discussion of whether more severe lack of funds in women of one faith versus another may affect service uptake. Also, our study population was relatively small and may not represent the wider North-Central region. Lastly, the study population was in North-Central communities. The findings may be different if Muslim women in the core North and Christian women in the South were interviewed; adherence to religious laws within the dominant religion may be stricter in those areas and therefore more dichotomy in views and influences may be unearthed.

## Recommendations for Future Research

Our study approach was to interview largely mixed groups of Christian and Muslim women together in the same focus groups. There may be added value for future studies to interview homogenous Muslim and Christian groups of women as well as male partners in separate groups with the objective of assessing the influence of religious beliefs and practices on maternal health service uptake. Socioeconomic status should also be taken into consideration when recruiting participants or collecting sociodemographic data. Studies exploring religious influences on maternal service uptake exclusively among HIV-positive women may provide valuable information for PMTCT programs. Qualitative studies targeting religious leaders themselves would also add strength to future study findings on religion and maternal service uptake.
